# Cross-group and longitudinal measurement invariance of the Child Eating Behavior Questionnaire-24G in middle childhood

**DOI:** 10.1186/s40337-026-01806-4

**Published:** 2026-10-27

**Authors:** Joyce C. Ruden, Petra Warschburger

**Affiliations:** Department of Psychology, Counseling Psychology, University of Potsdam, Potsdam, Germany

**Keywords:** Child Eating Behavior Questionnaire, Eating behavior, Childhood, Measurement invariance, Multigroup CFA, Group differences, Parent report

## Abstract

**Background:**

Childhood eating behaviors are most commonly assessed using parent-report questionnaires. To validly compare means across different groups or over time, equivalent item functioning is an important prerequisite. This study investigates the measurement invariance of a short German version of the Child Eating Behavior Questionnaire, the CEBQ-24G, across child sex, age, weight status, family socio-economic status, and parental weight status, as well as across three measurement occasions spanning three years in middle childhood.

**Methods:**

Analyses were conducted using a large community sample (*N* = 1,657) recruited from primary schools in Brandenburg, Germany. Measurement invariance was tested and latent group means were compared using Multigroup Confirmatory Factor Analysis (CFA).

**Results:**

The CEBQ-24G is measurement invariant on the residual level across all groups and measurement occasions, i.e., assessed differences in eating behaviors between these groups and measurement occasions are derived from actual differences in latent constructs and not a result of measurement bias—thus allowing valid comparisons of latent means across child sex, age, weight status, parental weight status, and family socio-economic status. We found significant differences in all group comparisons (except between age groups), although the eating behavior scales in which the differences in mean scores occurred varied depending on which groups were compared.

**Conclusions:**

Our findings suggest that the CEBQ-24G has good psychometric properties in all groups and is suitable for research into eating behaviors in middle childhood in diverse populations.

**Supplementary information:**

The online version contains supplementary material available at https://doi.org/10.1186/s40337-026-01806-4.

## Abstract (plain language summary)

The 24-item German adaptation of the Child Eating Behavior Questionnaire (CEBQ-24G) is a tool to assess a child’s eating behaviors via parent report. This study examined whether the questionnaire is understood in the same way across different groups and multiple measurements. Such measurement equivalence is required in order to make valid comparisons between eating behaviors across groups and over time. Using a sample of 1,657 children from primary schools in Brandenburg, Germany, we confirmed the questionnaire’s measurement equivalence across child sex, age, weight status, family socio-economic status, and parental weight status, as well as across time in middle childhood. This enabled us to compare eating behaviors across these groups. We found differences in eating behaviors based on children’s sex, weight status group, their parent’s weight status group, and their family’s socio-economic status, but not age. Overall, this study demonstrates that the CEBQ-24 validly assesses children’s eating behavior in diverse samples and over time.

**Supplementary information:**

The online version contains supplementary material available at https://doi.org/10.1186/s40337-026-01806-4.

## Introduction

It is widely recognized that children’s nutrition influences both their physical [1] and mental health [2, 3]. In recent years, researchers and healthcare professionals have increasingly focused on eating behaviors of children, as these traits not only shape what and how children eat from an early age [4], but also tend to remain stable into adolescence and adulthood [5, 6]. Research has shown that childhood eating behavior traits can differentiate diagnostic groups associated with overeating or food restriction during this developmental stage [7]. Moreover, they are consistently associated with childhood obesity [8], and predict eating disorder symptoms such as loss of control over eating, restrained eating, and compensatory behaviors later in life [9]. Consequently, childhood eating behaviors can serve as early indicators of potential nutritional challenges, and understanding these early dietary influences is crucial for developing effective prevention strategies for weight and eating disorders.

Based on Bronfenbrenner’s Ecological Systems Theory [10], Davison and Birch [11] developed a model of the development of childhood overweight, that describes influences on childhood eating behaviors from characteristics within the child (such as age or gender), to parenting styles and family characteristics (such as child feeding practices, parental food preferences, or parental weight status) as immediate influences; and to community, demographic, and societal characteristics (such as socio-economic status, school education programs, or accessibility of foods) as more distal influences. Similarly, the extensive DONE framework [12] aims to generate a comprehensive taxonomy of determinants of eating behavior across the life-span, using an interdisciplinary and multi-methodical approach. The determinants of nutrition and eating encompassed in the DONE framework range from factors within the individual (e.g., biological or psychological traits and states), to interpersonal (e.g., family structure or household socio-economic status), and finally to environmental and policy influences (e.g., food availability or governmental regulations).

To conduct meaningful research with regard to predictors of children’s eating behaviors, it is necessary to develop measurement instruments that adequately capture child eating behavior in populations that differ in these factors. Especially for questionnaires, which are the most commonly used and efficient way to gain insight into a person’s eating habits, this means that the included questionnaire items are understood and function equally in all target populations. Likewise, in order to examine longitudinal effects and developments in behaviors, measurements of these traits must be comparable between measurement points. Therefore, ensuring measurement equivalence is a crucial step in the development of a new measurement tool [13]. While psychometric equivalence of a measurement tool across specific populations allows researchers to compare group means and how group membership influences relations between constructs, its invariance over time is required for longitudinal analyses and comparisons between different measurements in the same sample [14]. Measurement invariance can be present at four increasingly restrictive levels [15]. The first level, configural invariance, requires that all items load onto the same corresponding factors across all groups or measurements, though factor loadings may differ. This ensures that the instrument measures similar constructs. The second level, metric invariance, requires equal factor loadings of items across all groups or measurements and allows for comparisons of regression slopes or change scores in longitudinal studies. Scalar measurement invariance is the third level and assumes equal intercepts across all groups or measurements and is required for comparing latent mean differences. The final level of measurement invariance additionally requires invariance of residuals across groups, and indicates that item differences between groups or measurements can be attributed to differences between groups or measurement occasions on the corresponding factors.

The Child Eating Behavior Questionnaire (CEBQ; [16]) is a 35-item comprehensive questionnaire intended to assess children’s eating behavior through parental report. The questionnaire measures children’s eating behavior across eight distinct dimensions, of which four measure the child’s tendency to seek out (Food Approach) and four measure the child’s tendency to avoid food (Food Avoidance). Due to its broad spectrum of assessed eating behaviors and ease of application, the CEBQ is one of the most used measurement instruments in childhood eating behavior research. There is, however, evidence that the CEBQ’s psychometric properties depend on sample characteristics [17]. Context and eating behavior norms might influence how parents understand CEBQ items and therefore influence their responses, causing factorial structures to vary across validation studies in different cultures or samples.

In the literature, various factors that influence eating behavior in children and parents’ interpretations thereof have been discussed, including intrapersonal factors such as the child’s sex [18], age [19], and weight status [8]. Furthermore, family characteristics such as socio-economic status or parental weight status have also been discussed as potential determinants of childhood eating [20]. Therefore, to investigate their influence on children’s eating behavior, it is necessary to establish measurement invariance of the CEBQ across groups that differ in these traits. However, studies investigating measurement invariance of the CEBQ across demographic groups remain scarce. In a validation study for the Chinese translation of the CEBQ, Zhou and Sun [21] confirmed full scalar measurement invariance for the CEBQ in boys and girls. Domoff et al. [22] reported scalar measurement invariance across White and Black non-Hispanic participants. Perez et al. [23] examined the measurement equivalence of each CEBQ subscale individually across sex, ethnicity, and household food security. While the “Enjoyment of Food”, “Emotional Overeating”, and “Satiety Responsiveness” performed equally across the above groups, “Food Fussiness”, “Desire to Drink”, “Slowness in Eating”, and “Emotional Undereating” varied significantly—especially between food-secure and food-insecure households. However, Perez et al. only examined measurement invariance of individual subscales, and did not test for measurement invariance of the entire questionnaire’s factor structure. To our knowledge, measurement invariance across child age and weight status, family socio-economic status, or parental weight status has not yet been examined.

Recently, we investigated the psychometric properties of a 24-item short version of the CEBQ for use in German populations, the CEBQ-24G [24]. In this previous validation study, we confirmed the expected eight-factor structure of the adapted questionnaire with three items per factor through both exploratory and confirmatory factor analysis. The model metrics indicated a good fit (RMSEA = 0.064 [90% CI 0.060–0.068], CFI = 0.910, SRMR = 0.098, χ2(243) = 1183.050, *p* < .001). All subscales demonstrated satisfactory internal consistencies, with McDonald’s ω ranging between 0.72 for Satiety Responsiveness to 0.90 for Desire to Drink. Moreover, we observed the expected correlations with children’s BMI standardized for age and sex, as well as scores on Restrained and External Eating (DEBQ-K [25]), confirming the questionnaire’s convergent validity. The aim of the current study is to extend the results of this validation by testing for measurement invariance across groups that differ in key aspects that might influence children’s eating behavior or parents’ interpretation thereof: child sex, age, and weight status, parental weight status, and family socio-economic status. Given that measurement invariance between these groups can be confirmed, we also aim to compare latent group means to identify significant differences in eating behavior based on these traits. Moreover, we aim to investigate whether the CEBQ-24G’s factorial structure is stable over time and allows for longitudinal comparisons by investigating measurement invariance across three subsequent measurement waves.

## Methods

### Procedure and participants

The current study uses data from the PIER-study, a larger ongoing research project focusing on risk factors and commonly observed developmental issues in childhood and adolescence [26]. In 2011 (T1), 1,657 children aged 6–11 years (M = 8.36, SD = 0.95) and their parents were recruited from primary schools in the federal state of Brandenburg, Germany. Follow-up measurements were conducted approximately 12 months (T2) and 36 months (T3) later. At T1, caregivers provided CEBQ-24G data for 1,337 children. At T2 and T3, data were provided for 1,193 children and 1,063 children, respectively. Table 1 provides an overview of the T1 sample characteristics.

**Table 1:** Sample description

Age (years)	*N* (%)
6–7	119 (8.90)
8–9	841( 62.90)
10–11	375 ( 28.05)
Missing	2 (0.15)
**Child Sex**	
Girls	691 (51.68)
Boys	646 (48.32)
**Child Weight Status** ^**1**^	
Underweight (< 15.50th percentile)	88 (6.58)
Normal Weight (15.50th – 90.49th percentile)	998 (74.64)
Overweight (90.50th – 98.90th percentile)	172 (12.86)
Obesity (> 98.90th percentile)	67 (5.01)
Missing	12 (0.90)
**Reporter**	
Mother	932 (69.71)
Father	97 (7.26)
Both Parents	273 (20.42)
Other Caregiver	12 (0.90)
Missing	23 (1.72)
**Weight Status of Reporting Parent** ^**2**^	
Underweight (BMI < 18.50)	19 (1,42)
Normal Weight (BMI 18.50-24.99)	640 (47,87)
Overweight (BMI 25.00-29.99)	246 (18,40)
Obesity (BMI ≥ 30.00)	101 (7,55)
Missing	331 (24,76)
**Highest Parental Education Degree** ^**3**^	
None	9 (0.67)
Special education certificate	5 (0.37)
Lower secondary education (Hauptschulabschluss)	33 (2.47)
Intermediate secondary education (Realschulabschluss)	420 (31.41)
Higher education entrance qualification (Fachhochschulreife/Abitur)	257 (19.22)
Higher education degree (Fachhochschul-/Hochschulabschluss)	607 (45.40)
Missing	6 (0.40)

Note: T1 data, *N* = 1,337. ^1^Children’s weight status was determined based on international IOTF-cut-offs [27]. ^2^Parental weight status was determined based on WHO’s recommended cut-off values [28], cases where both parents completed the questionnaire together or the reporter was a caregiver other than mother or father (*n* = 308) were coded as missing. ^3^Parents’ highest educational qualification refers to the German education system, equivalent degrees are assigned to the corresponding category

### Measures

#### Demographics

Children’s sex and date of birth were assessed during in-school interviews; children’s age at time of assessment was then calculated to the day. Children’s height and weight were obtained by trained personnel using calibrated digital scales and ultrasound measurement devices, after removal of shoes and heavy outerwear. After calculation of BMI, weight status categorization for children was based on international IOTF cut-offs [27].

Parents self-reported their demographic data. Family socio-economic status was operationalized via the parents’ highest achieved educational qualification. Parents’ highest educational attainment was used as a proxy for socio-economic status, as higher levels of educational attainment are generally associated with better employment opportunities and higher earnings [29, 30]. BMI was calculated based on self-reported height and weight, and parental weight status was classified based on WHO’s recommended cut-off values [28].

#### CEBQ-24G

Parents were asked to report their children’s eating behavior using the CEBQ-24G, a translated 24-item version of the original CEBQ that assesses the eight dimensions of eating behavior with three items per scale. The Food Responsiveness scale assesses a child’s tendency to react to food-related stimuli. “Enjoyment of Food” is defined as the hedonistic pleasure derived by the child from eating. “Desire to Drink” is defined as the demand for sweetened beverages. “Emotional Overeating” and “Emotional Undereating” measure the influence of emotional states on the child’s eating. “Slowness in Eating” is defined as the speed at which a child tends to eat. “Satiety Responsiveness” is defined as the child’s reaction to their body’s own signals of feeling full. “Food Fussiness” assesses the child’s reaction to unknown or disliked foods. Food Responsiveness, Enjoyment of Food, Desire to Drink, and Emotional Overeating are grouped under Food Approach scales and measure the child’s tendency to seek out food. Slowness in Eating, Satiety Responsiveness, Food Fussiness, and Emotional Undereating are grouped under Food Avoidance scales, measuring the child’s inclination to avoid food and eating. Responses were coded on a 5-point Likert scale ranging from “never” to “always.” The CEBQ-24G showed good psychometric properties in a previous validation study [24].

### Statistical analyses

Analyses were conducted using *R Statistical Software* (version 4.4.1; [31]), *RStudio* (version 2024.9.0.375; [32]), and the *lavaan*-package [33]. Measurement invariance across child sex, age, weight status, parental weight status, and family socio-economic status at T1 was tested using multigroup CFA [15]. In order to achieve an adequate sample size for all groups, child and parental weight status were classified into two categories (under-/normal weight and overweight/obese) based on cut-offs recommended by the IOTF for children [27], and the WHO [34] for adults. Child age was categorized into three groups: ages 6–7, ages 8–9, and ages 10–11.

To ensure correct categorization of the reporting caregiver, cases in which a person other than the mother or father of a child provided data or in which both parents filled out the questionnaire together were excluded from this analysis. A similar procedure was followed concerning family socio-economic status. Participants were divided into two groups based on parents’ highest educational qualification: Group 1 was comprised of families in which both parents reported ten or fewer years of school education, while Group 2 consisted of families in which at least one parent reported either higher education entrance qualification or a degree from university or technical college. This distinction reflects an important educational threshold in Germany, as the Abitur provides access to higher education and occupational opportunities usually associated with higher income. The grouping was thus intended to capture differences in socio-economic resources associated with educational attainment.

**Fig. 1:**
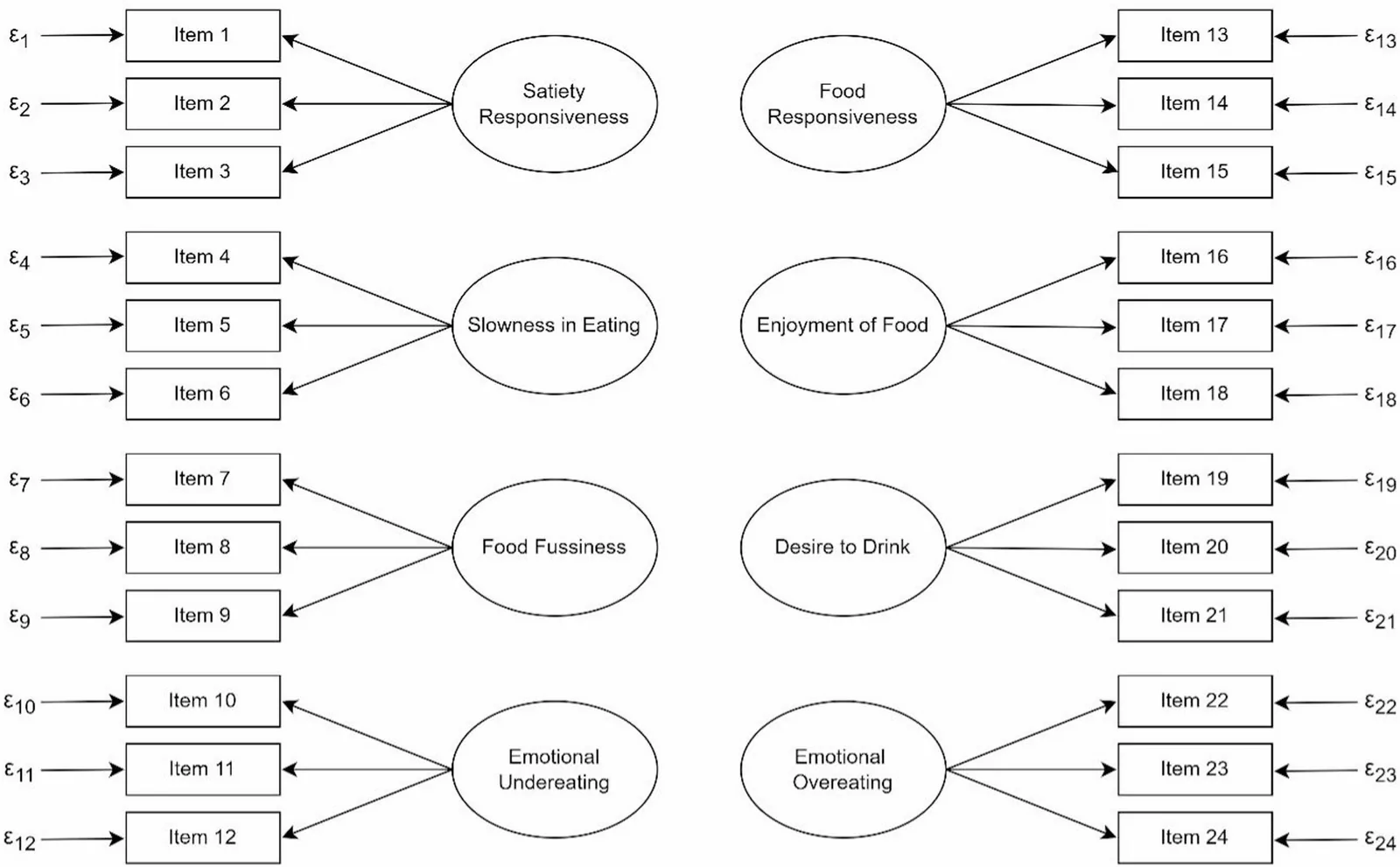
Factor structure of the CEBQ-24G. Correlations between factors are omitted for clarity.

In a first step, the previously established eight-factor model of the CEBQ-24G (see Fig. 1) was applied to all subsamples. Since χ^2^ is sensitive to sample size, model fit was evaluated adhering to established model fit indices recommended by Keith [35]: CFI ≥ 0.95, RMSEA ≤ 0.05, and SRMR ≤ 0.06 indicate a good model fit, while CFI ≥ 0.90, RMSEA ≤ 0.08, and SRMR ≤ 0.08 indicate an acceptable model fit. If the model fit is considered to be at least acceptable (indicating configural invariance), restrictions in factor loadings, intercepts, and residuals are applied to both groups for testing measurement invariance. Chen [15] suggests using changes in CFI, RMSEA, and SRMR between increasingly restrictive models to test measurement invariance. In line with Chen’s recommendations, the following cut-off values were implemented: For assessing loading invariance, a change greater than 0.010 in CFI, along with a change greater than 0.015 in RMSEA or a change greater than 0.030 in SRMR (greater than 0.010 for testing intercept or residual invariance), indicates lack of invariance. All multigroup CFAs were estimated using Maximum Likelihood with robust standard errors and Satorra-Bentler scaled test statistic [36], the factor loadings of the first items were set to 1.

To determine potential differences in eating behaviors between groups, we compared latent means if at least scalar measurement invariance could be established. Latent mean scores in the first group were fixed to zero, and significant mean differences between groups were signified by a p-value lower than 0.05 or by no overlaps in 95% confidence intervals for subsequent groups.

Finally, to ensure temporal stability of the CEBQ-24G’s factorial structure, measurement invariance across three measurement waves was tested following the procedure outlined by Mackinnon et al. [37]. The assumption of measurement invariance across the three measurement occasions was tested by applying the same guidelines as described above. For this analysis, we included the total sample of 1,466 children whose parents provided CEBQ-24G data at least once during the three measurement occasions.

## Results

### Missing data

The amount of missing data in individual CEBQ-24G items was generally low at each measurement occasion (0,1–1,6%; see Appendix A, Table A). However, there was some notable drop-out between the measurement occasions (18.25% between T1 and T2, and 20.20% between T2 and T3). Hence, we conducted binomial logistic regression to examine whether non-participation at subsequent measurements was systematically associated with observed variables. Predictors included the CEBQ-24G subscales, child age, child BMI standardized for age and sex, child sex, the highest parental education status as a proxy for family socio-economic status, and BMI of both parents.

For the transition from T1 to T2, a significant association was found between family socio-economic status and non-participation at T2 (*b* = -0.358, *p* < .001), with households with a higher parental education status being more likely to participate. No significant associations were observed for the other predictors included in the model.

For the transition from T2 to T3, family socio-economic status was again significantly associated with non-participation (*b* = -0.353, *p* < .001). Moreover, older child age (*b* = 0.231, *p* = .021) was associated with a higher likelihood of non-participation at T3, whereas higher maternal BMI (*b* = -0.054, *p* = .025) and higher scores on Food Fussiness at T2 (*b* = -0.196, *p* = .050) were associated with a lower likelihood of non-participation at T3.

Overall, these findings suggest that drop-out was not completely random across measurement occasions. Hence, we accounted for missing data in the ultigroup CFA using Full Information Maximum Likelihood (FIML) which is recommended in this case as it provides unbiased parameter estimates under the assumption that data are Missing at Random instead of Missing Completely at Random [38].

### Measurement invariance

Figure 1 shows the eight-factor structure of the CEBQ-24G, which was applied in multigroup CFAs across the respective grouping variables. Fit indices of the respective models at the increasingly restrictive levels of invariance testing are presented in Table 2. Fit indices indicated an at least acceptable fit for the eight-factor structure in all subsamples; and according to Chen’s [15] guidelines for testing measurement invariance, changes in fit indices support residual invariance across child sex, child weight status, parental weight status, and family socio-economic status. The difference in CFI for testing residual invariance across child weight status and family socio-economic status surpassed the threshold of 0.010 slightly; however, since these changes were only marginally above the defined cut-off values and were not accompanied by substantial changes in RMSEA or SRMR, we decided to assume residual measurement invariance nevertheless.

**Table 2:** Measurement invariance across groups

Model	χ^2^	df	CFI	RMSEA	90%CI	SRMR	Model Comparison	Δχ^2^	Δdf	ΔCFI	ΔRMSEA	ΔSRMR
**Child Sex**												
Girls (*N* = 691)	608.205	224	0.942	0.052	0.047, 0.057	0.056						
Boys (*N* = 646)	595.930	224	0.948	0.051	0.046, 0.056	0.051						
Configural	1204.497	448	0.945	0.052	0.048, 0.055	0.054						
Metric	1235.686	464	0.943	0.052	0.048, 0.055	0.056	Configural vs. Metric	31.189	16	-0.002	0.000	0.002
Scalar	1273.222	480	0.942	0.051	0.048, 0.055	0.056	Metric vs. Scalar	37.536	16	-0.001	-0.001	0.000
Residual	1242.368	504	0.942	0.050	0.046, 0.054	0.056	Scalar vs. Residual	-30.854	24	0.000	-0.001	0.000
**Child Age**												
6–7 years (*N* = 119)	359.123	224	0.913	0.065	0.050, 0.080	0.076						
8–9 years (*N* = 841)	685.854	224	0.946	0.052	0.047, 0.056	0.053						
10–11 years (*N* = 375)	423.242	224	0.952	0.048	0.041, 0.056	0.054						
Configural	1490.025	672	0.945	0.052	0.048, 0.056	0.056						
Metric	1494.890	704	0.945	0.051	0.047, 0.055	0.057	Configural vs. Metric	4.865	32	0.000	-0.001	0.001
Scalar	1522.688	736	0.945	0.050	0.046, 0.053	0.057	Metric vs. Scalar	27.798	32	0.000	-0.001	0.000
Residual	1564.958	784	0.941	0.047	0.044, 0.051	0.059	Scalar vs. Residual	42.270	48	-0.004	-0.003	0.002
**Child Weight Status**												
Under- and Normal Weight (*N* = 1086)	758.713	224	0.947	0.049	0.045, 0.053	0.049						
Overweight and Obesity (*N* = 239)	395.435	224	0.937	0.056	0.046, 0.065	0.064						
Configural	1163.944	448	0.945	0.050	0.046, 0.054	0.052						
Metric	1213.738	464	0.942	0.051	0.047, 0.054	0.054	Configural vs. Metric	49.794	16	-0.003	0.001	0.002
Scalar	1308.794	480	0.936	0.052	0.049, 0.056	0.054	Metric vs. Scalar	95.056	16	-0.006	0.001	0.000
Residual	1467.658	504	0.921	0.057	0.053, 0.060	0.057	Scalar vs. Residual	158.864	24	-0.015	0.005	0.003
**Parental Weight Status**												
Under- and Normal Weight (*N* = 659)	640.789	224	0.937	0.055	0.050, 0.060	0.057						
Overweight and Obesity (*N* = 347)	511.699	224	0.927	0.060	0.053, 0.068	0.057						
Configural	1155.397	448	0.934	0.057	0.052, 0.061	0.057						
Metric	1157.346	464	0.933	0.056	0.052, 0.060	0.058	Configural vs. Metric	1.949	16	-0.001	-0.001	0.001
Scalar	1183.107	480	0.933	0.055	0.051, 0.059	0.058	Metric vs. Scalar	25.761	16	0.000	-0.001	0.000
Residual	1192.369	504	0.930	0.055	0.051, 0.059	0.060	Scalar vs. Residual	9.262	24	-0.003	0.000	0.002
**Family Socio-Economic Status**												
Low (*N* = 467)	457.244	224	0.953	0.048	0.041, 0.055	0.055						
High (*N* = 864)	707.112	224	0.946	0.051	0.047, 0.055	0.052						
Configural	1162.747	448	0.948	0.050	0.046, 0.054	0.053						
Metric	1190.916	464	0.946	0.050	0.046, 0.054	0.054	Configural vs. Metric	28.169	16	-0.002	0.000	0.001
Scalar	1242.536	480	0.944	0.050	0.047, 0.054	0.055	Metric vs. Scalar	51.620	16	-0.002	0.000	0.001
Residual	1364.775	504	0.934	0.053	0.050, 0.057	0.056	Scalar vs. Residual	122.239	24	-0.010	0.003	0.001

Note: CFI = Comparative Fit Index; RMSEA = Root Mean Square Error of Approximation; SRMS = Standardized Root Mean Square Residual

The results of the measurement invariance testing across all grouping variables allow mean comparisons of eating behaviors between these groups. Means were calculated in multigroup CFA with factor loadings, intercepts, and residuals equalized. The results of all latent mean comparisons are listed in detail in Appendix A, Table B. There were significant differences between boys and girls in Food Responsiveness, Slowness in Eating, and Food Fussiness, with boys scoring lower in Food Responsiveness and Slowness in Eating, and higher in Food Fussiness. Children in the combined overweight and obesity category scored significantly higher in all Food Approach scales and lower in Satiety Responsiveness and Slowness in Eating than children in the under- and normal weight category, with no significant difference in Food Fussiness and Emotional Undereating between these groups. We found no significant mean differences between age groups. Parents in the overweight or obesity category rated their children as having significantly higher Food Responsiveness and Desire to Drink than did parents in the under- and normal weight category. Finally, children in families categorized in the higher socio-economic status category scored lower in Food Responsiveness and Desire to Drink and higher in Emotional Undereating than children in the lower socio-economic status category.

Lastly, we tested for measurement invariance across three measurement points to establish temporal stability of the CEBQ-24G. Fit indices of the four models can be found in Table 3. The decrease in fit indices did not surpass the recommended thresholds at any level of restrictions, supporting residual measurement invariance across three measurements.

**Table 3:** Measurement invariance across measurement waves

Model	χ^2^	df	CFI	RMSEA	90%CI	SRMR	Model Comparison	Δχ^2^	Δdf	ΔCFI	ΔRMSEA	ΔSRMR
T1 (*N* = 1337)	928.490	224	0.947	0.051	0.047, 0.054	0.050						
T2 (*N* = 1193)	868.132	224	0.948	0.051	0.048, 0.055	0.052						
T3 (*N* = 1063)	757.852	224	0.953	0.049	0.045, 0.053	0.057						
Configural	4059.462	2184	0.962	0.028	0.026, 0.029	0.048						
Metric	4181.640	2232	0.960	0.028	0.027, 0.030	0.051	Configural vs. Metric	122.178	48	-0.002	0.000	0.003
Scalar	4527.125	2280	0.953	0.030	0.029, 0.031	0.053	Metric vs. Scalar	345.485	48	-0.007	0.002	0.002
Residual	4585.961	2328	0.952	0.030	0.029, 0.032	0.053	Scalar vs. Residual	58.836	48	-0.001	0.000	0.000

Note: CFI = Comparative Fit Index; RMSEA = Root Mean Square Error of Approximation; SRMS = Standardized Root Mean Square Residual

## Discussion

Childhood eating behaviors are complex and are influenced by a number of factors, both within the child and in their environment. The Childhood Eating Behavior Questionnaire [16] and our 24-item adaptation for the German language (CEBQ-24G [24]), assess eating behavior traits in children on eight distinct subscales. However, in order to reliably investigate influences on eating behaviors and make comparisons between groups or across multiple measurement occasions, it is necessary to first establish measurement invariance. Despite the questionnaire’s popularity in eating behavior research, studies investigating measurement invariance across populations or measurements remain scarce. Therefore, our aim was to investigate measurement invariance of the CEBQ-24G across groups that differ in properties that have been discussed as influencing children’s eating behaviors.

The CEBQ-24G proves to be measurement invariant on the residual level across child sex, weight status, and age group, as well as across parental weight status and family socio-economic status. On a practical level, this means that across these groups, the CEBQ-24 measures the same constructs and differences in means, and variances of the observed scores are thus the result of differences in latent traits, which supports the applicability of the CEBQ-24G to diverse samples of German-speaking parents with primary school-aged children. With regard to child sex, we were therefore able to replicate findings by Zhou and Sun [21], who also reported strict measurement invariance across boys and girls for the Chinese adaptation of the CEBQ. As of today, measurement invariance across the other groups has not yet been subject to study. However, we could not find measurement bias across child weight, which we expected since Manzano et al. [39] and Nigam et al. [40] were unable to reproduce the original eight-factor structure of the CEBQ in samples of children with overweight and obesity. We could also establish residual measurement invariance across three measurement occasions conducted over three years, allowing for longitudinal analyses and interpretation of developmental trends in middle childhood.

Since at least scalar measurement invariance was given across all tested groups, we conducted latent mean comparisons. Overall, we observed only a limited number of sex-related differences in eating behaviors. We found that boys scored significantly lower in Food Responsiveness and Slowness in Eating, and higher in Food Fussiness than girls. This means that within our sample, parents report boys to eat faster, to accept only a narrower range of food, and to be less likely to seek out food in response to food cues than girls. Sex differences in CEBQ subscales have been investigated in similarly aged samples before (e.g [16, 41–43]), , but results have been inconsistent across studies. In their review on sex differences in childhood eating behaviors, Keller et al. [18] conclude that sex differences in eating behaviors are more often found in school-aged than in preschool children and likely result from parental feeding practices and social influences. For instance, the greater acceptance of less-energy-dense foods such as vegetables and fruit in girls might be a result of a higher societal pressure for females to attain a thin body ideal. This pressure promotes the societal idea that women should eat smaller portions and healthier foods. It is possible that the differences in eating behavior between boys and girls become even more pronounced during adolescence, when physiological changes occur and the influence of sociocultural factors on children further increases. However, since existing evidence suggests that such differences may also manifest in earlier developmental stages, there is a need for further research on sex differences in eating behaviors.

Research on differences between child weight status groups has been more consistent, with children categorized as overweight or obese scoring higher in Food Approach subscales and lower in Food Avoidance subscales in the majority of studies [8]. Our results are in line with the previous literature, and the differences in eating behavior traits were most consistent and prominent between weight status groups. The CEBQ was originally developed to identify eating styles that are associated with the development of overweight or underweight in children within the Behavioral Susceptibility Theory framework [16]. Therefore, differences between weight status groups were in line with theoretical considerations. However, we did not observe significant differences in Food Fussiness and Emotional Undereating. This aligns with the conclusions by Kininmonth et al. [8] who report that associations between these subscales and child weight have been inconsistent in previous studies. Furthermore, we also did not observe significant correlations between Food Fussiness or Emotional Undereating and BMI-scores standardized for age and sex in our previous validation study for the CEBQ-24G [24]. Food Fussiness and Emotional Undereating therefore do not seem to be linked to child weight status like the other subscales of the CEBQ-24G.

We did not find significant differences in eating behaviors between children aged 6–7, 8–9, or 10–11. While some previous studies did report mean differences between age groups (e.g., [16, 43, 44]), most of these studies either examined younger children or samples with a wider age range. It is possible that changes in eating behaviors manifest more prominently during the transitions from infancy to childhood (e.g., [45]), and from childhood to adolescence (e.g., [42]), while the middle childhood is marked by relative stability. Our results are in line with Sleddens et al. [46] who compared eating behavior scores between a group of six-year-olds and a group of seven-year-olds and also found no significant age differences. This could provide an explanation for why the differences in eating behavior traits between the age groups represented in our sample did not reach statistical significance.

Mean differences based on parental weight has so far only been investigated in two prior studies, which found either no differences [47] or a significant difference in Food Fussiness [48] between parental weight status groups. We could not replicate these results in our sample, since our mean comparisons showed that children whose parents were categorized in the upper weight category scored significantly higher in both Food Responsiveness and Desire to Drink, not in Food Fussiness. However, the previous studies were both conducted using younger samples. Previous research shows that overweight mothers worry more about their children’s weight status, regardless of the children’s current weight [49]. It is possible that these concerns become more salient in school-aged children, causing parents with higher weight to become more observant of Food Approach behaviors such as eating in response to external food cues and consumption of sweetened beverages which are associated with weight gain. However, overall, we found only a limited number of differences between parental weight status groups, indicating that the parent’s weight status might not be as influential on childhood eating as other factors.

Finally, we found that children in the upper socio-economic status group – indicated by parental education status – scored significantly lower on Food Responsiveness and Desire to Drink, and higher on Emotional Undereating. So far, mean differences based on socio-economic status groups have not yet been investigated. However, since lower socio-economic status has been associated with poorer diet quality [50–52] and higher BMI [53, 54] in previous studies, these results may be linked to higher scores in Food Approach traits in children with lower family socio-economic status.

### Strengths and limitations

While our findings provide important insights into the psychometric properties of the CEBQ-24G in different groups and across multiple measurements, it is essential to contextualize them by considering the strengths and limitations of the present study. As a strength, it should be noted that we conducted our analyses using a large community sample (*N* = 1,657) of German primary school children and their parents and were able to establish residual measurement invariance across all tested groups, thereby ensuring that the results of our comparisons of eating behavior trait means are derived from actual differences in latent constructs between these groups and measurement occasions rather than measurement bias. Children’s weight status categorization was based on measured height and weight and standardized for age and sex, which is more reliable than parent- or self-reporting [55].

However, due to the nature of our community sample, the results of our analyses should also be interpreted considering some limitations. Unlike the child’s weight status, parental weight status was based on self-reported data which carries the risk of inaccuracy. Nevertheless, particularly in large-scale studies, these data are considered valuable information and useful indicators [56]. Moreover, our measure of socio-economic status cannot properly address food security within the family, which has been argued as a possible influence factor on the factorial structure of the CEBQ in previous studies [23]. We were able to test measurement invariance across child sex, but since the CEBQ-24G is a parent-reported measure, the sex of the parent providing information of their child’s eating behaviors might also influence their understanding of CEBQ-24G items. However, as in many previous studies on child eating behavior, data was mainly provided by mothers [57], with too few fathers’ reports (5.84%) to conduct measurement invariance testing across parental sex. This should be addressed in future studies, since fathers also influence and observe children’s eating and should be considered in eating behavior research [58, 59].

## Conclusion

Our work demonstrates that the CEBQ-24G proves to be measurement invariant across child sex, age, weight status, family socio-economic status, and parental weight status. This allowed us to validly compare latent means across these groups and is a crucial prerequisite for future analyses aimed at identification of influences on childhood eating behaviors. Moreover, the CEBQ-24G is measurement invariant across three measurement occasions spanning three years of middle childhood, allowing for longitudinal analyses on changes in eating behaviors during this time. Since childhood eating behavior traits can serve as indicators of current or future disordered eating [7, 9], these findings are significant for future studies in eating disorder research. Establishing cross-group measurement equivalence is essential for the CEBQ-24G’s application in diverse samples and for making valid comparisons between target groups. This approach may help identify children at risk of developing future eating- or weight-related issues. Similarly, ensuring longitudinal measurement invariance allows researchers to track changes in eating behaviors over time, which is particularly important for assessing the effectiveness of interventions aimed at modifying eating behavior traits in children, since behavioral weight management programs have shown potential to instigate such changes [60]. However, future research is needed to ensure equivalent item functioning in other populations, e.g., with respect to different age groups, low family socio-economic status, or reports on childhood eating behaviors given by fathers or other caregivers.

## Supplementary information

Supplementary Material 1

## Data Availability

The datasets generated and analyzed during the current study are not publicly available, as the participants were not asked to consent to publication within repositories but are available from the corresponding author upon reasonable request.

## Abbreviations

BMI: Body-Mass-Index
CFA: Confirmatory Factor Analysis
CFI: Comparative Fit Index
IOTF: International Obesity Task Force
RMSEA: Root Mean Square Error of Approximation
SRMR: Standardized Root Mean Square Residual
WHO: World Health Organization
