# Supplementary material for: Cross-group and longitudinal measurement invariance of the Child Eating Behavior Questionnaire-24G in middle childhood
Source: J Eat Disord. 2026 Oct 27;14:252. doi: 10.1186/s40337-026-01806-4 (PMC13711984; doi:10.1186/s40337-026-01806-4)
Supplement: Supplementary file 1 [file 40337_2026_1806_MOESM1_ESM.pdf]

Appendix A

Table A: *Sample Size (N) per Item*

Subscale and Items	T1	T2	T3
**Satiety Responsiveness**			
1. Mein Kind isst seinen Teller nicht ganz leer/*My child leaves food on his/her plate at the end of a meal*	1333	1186	1061
2. Mein Kind ist satt, bevor es aufgegessen hat/*My child gets full before his/her meal is finished*	1330	1188	1057
3. Mein Kind kann keine Mahlzeit essen, wenn es kurz zuvor eine Kleinigkeit gegessen hat/*My child cannot eat a meal if his/he has had a snack just before*	1330	1187	1059
**Slowness in Eating**			
4. Mein Kind isst langsam/*My child eats slowly*	1334	1187	1062
5. Mein Kind isst seinen Teller sehr schnell leer/*My child finishes his/her meal very quickly*	1335	1186	1061
6. Mein Kind isst während des Essens zunehmend langamer/*My child eats more and more slowly during the course of a meal*	1328	1182	1056
**Food Fussiness**			
7. Meinem Kind gefällt es, neues Essen auszuprobieren/*My child enjoys tasting new foods*	1335	1188	1061
8. Mein Kind lehnt unbekanntes Essen zuerst ab/*My child refuses new foods at first*	1333	1185	1061
9. Mein Kind entscheidet, dass ihm etwas nicht schmeckt, auch ohne es vorher probiert zu haben/*My child decides that s/he doesn't like food, even without tasting it*	1329	1187	1061
**Emotional Undereating**			
10. Mein Kind isst weniger, wenn es aufgebracht ist/*My child eats less when s/he is upset*	1326	1177	1056
11. Mein Kind isst weniger, wenn es wütend ist/*My child eats less when s/he is angry*	1324	1175	1046
12. Mein Kind isst mehr, wenn es fröhlich ist/*My child eats more when s/he is happy*	1322	1178	1050
**Food Responsiveness**			
13. Wenn mein Kind könnte, hätte es immer etwas zu Essen im Mund/*Given the chance, my child would always have food in his/her mouth*	1334	1185	1061
14. Wenn es die Wahl hätte, würde mein Kind die meiste Zeit essen/*Given the choice, my child would eat most of the time*	1335	1185	1052
15. Wenn mein Kind dürfte, würde es zu viel essen/*If allowed to, my child would eat too much*	1331	1181	1060
**Enjoyment of Food**			
16. Mein Kind isst gern/*My child enjoys eating*	1327	1185	1060
17. Mein Kind liebt Essen/*My child loves food*	1328	1187	1058

18.	Mein Kind freut sich auf die Essenszeiten/*My child looks forward to meal times*	1329	1181	1055
**Desire to Drink**				
19.	Wenn mein Kind könnte, würde es ständig trinken/*If given the chance, my child would always be having a drink*	1331	1182	1059
20.	Wenn mein Kind könnte, würde es den ganzen Tag trinken/*If given the chance, my child would drink continuously throughout the day*	1330	1183	1056
21.	Mein Kind fragt ständig nach etwas zu trinken/*My child is always asking for a drink*	1330	1187	1059
**Emotional Overeating**				
22.	Mein Kind isst mehr, wenn es ängstlich ist/*My child eats more when anxious*	1323	1176	1053
23.	Mein Kind isst mehr, wenn es verärgert ist/*My child eats more when annoyed*	1325	1177	1054
24.	Mein Kind isst mehr, wenn es besorgt ist/*My child eats more when worried*	1321	1174	1048

Table B: *Estimated Differences in Latent Means*

**Child Sex (Reference Group: Girls)**					
Boys:					
Factor	Mean Difference	Std. Error	p	Lower 95% CI	Upper 95% CI
Food Resp.	-0.126	0.053	0.018	-0.230	-0.022
Enjoyment of Food	-0.103	0.062	0.100	-0.225	0.020
Desire to Drink	0.055	0.061	0.366	-0.064	0.174
Emot. Overeating	-0.093	0.055	0.087	-0.200	0.013
Slowness in Eating	-0.245	0.070	< 0.001	-0.381	-0.108
Satiety Resp.	-0.101	0.065	0.121	-0.227	0.026
Food Fussiness	0.161	0.059	0.006	0.046	0.276
Emot. Undereating	-0.021	0.062	0.739	-0.142	0.100
**Child Age (Reference Group: 6-7 years)**					
8-9 years:					
Factor	Mean Difference	Std. Error	p	Lower 95% CI	Upper 95% CI
Food Resp.	0.155	0.125	0.213	-0.089	0.400
Enjoyment of Food	0.142	0.109	0.191	-0.071	0.355
Desire to Drink	-0.103	0.096	0.282	-0.291	0.085
Emot. Overeating	0.258	0.162	0.110	-0.059	0.575
Slowness in Eating	-0.052	0.118	0.657	-0.283	0.179
Satiety Resp.	-0.152	0.112	0.177	-0.372	0.069
Food Fussiness	-0.037	0.107	0.726	-0.248	0.173
Emot. Undereating	-0.089	0.105	0.396	-0.296	0.117
10-11 years:					
Factor	Mean Difference	Std. Error	p	Lower 95% CI	Upper 95% CI
Food Resp.	0.236	0.145	0.105	-0.049	0.521
Enjoyment of Food	0.123	0.119	0.301	-0.110	0.356
Desire to Drink	-0.091	0.103	0.375	-0.293	0.111
Emot. Overeating	0.322	0.180	0.074	-0.031	0.674
Slowness in Eating	-0.205	0.129	0.112	-0.458	0.048
Satiety Resp.	-0.236	0.122	0.054	-0.475	0.004
Food Fussiness	-0.083	0.117	0.479	-0.313	0.147
Emot. Undereating	-0.044	0.114	0.701	-0.268	0.180
**Child Weight Status (Reference Group: Lower Weight Status)**					
Higher Weight Status:					
Factor	Mean Difference	Std. Error	p	Lower 95% CI	Upper 95% CI
Food Resp.	1.485	0.169	< 0.001	1.154	1.817
Enjoyment of Food	0.662	0.081	< 0.001	0.504	0.820
Desire to Drink	0.397	0.083	< 0.001	0.235	0.559
Emot. Overeating	0.471	0.121	< 0.001	0.234	0.709
Slowness in Eating	-0.476	0.093	< 0.001	-0.657	-0.294
Satiety Resp.	-0.568	0.080	< 0.001	-0.724	-0.412
Food Fussiness	-0.106	0.075	0.159	-0.253	0.041
Emot. Undereating	-0.065	0.077	0.397	-0.215	0.085

**Parental Weight Status (Reference Group: Lower Weight Status)**					
Higher Weight Status:					
Factor	Mean Difference	Std. Error	p	Lower 95% CI	Upper 95% CI
Food Resp.	0.280	0.092	0.002	0.100	0.460
Enjoyment of Food	0.100	0.072	0.166	-0.041	0.241
Desire to Drink	0.184	0.076	0.015	0.036	0.332
Emot. Overeating	0.093	0.084	0.268	-0.072	0.258
Slowness in Eating	-0.063	0.080	0.430	-0.221	0.094
Satiety Resp.	-0.020	0.076	0.792	-0.170	0.129
Food Fussiness	-0.021	0.073	0.770	-0.164	0.122
Emot. Undereating	-0.031	0.074	0.676	-0.176	0.114
**Family Socio-Economic Status (Reference Group: Lower Family Socio-Economic Status)**					
Higher Family Socio-Economic Status:					
Factor	Mean Difference	Std. Error	p	Lower 95% CI	Upper 95% CI
Food Resp.	-0.238	0.048	< 0.001	-0.332	-0.144
Enjoyment of Food	0.048	0.061	0.430	-0.071	0.167
Desire to Drink	-0.438	0.051	< 0.001	-0.537	-0.338
Emot. Overeating	-0.093	0.057	0.101	-0.204	0.018
Slowness in Eating	-0.013	0.069	0.856	-0.148	0.123
Satiety Resp.	0	0.066	0.995	-0.129	0.128
Food Fussiness	-0.052	0.063	0.411	-0.175	0.071
Emot. Undereating	0.268	0.074	< 0.001	0.123	0.413

Note. Resp. = Responsiveness, Emot. = Emotional
